# Osteochondroma of tibial tuberosity in a young soccer player mimicking an Osgood-Schlatter disease: A case report and literature review

**DOI:** 10.1016/j.radcr.2025.05.004

**Published:** 2025-05-24

**Authors:** Roberto Calbi, Angela Ventura, Annachiara Ceccherini, Roberta Dattoli, Mario Di Diego, Diletta De Lucia, Fabio Laterza, Michele Dezio, Raffaele Garofalo

**Affiliations:** a Radiology Unit, Ente Ecclesiastico Ospedale Generale Regionale "F.Miulli", Acquaviva delle Fonti, Bari, Italy; b Department of Radiology, G.B. Rossi Hospital, University of Verona, Verona, Italy; c Department of Diagnostic Imaging, Fondazione Policlinico Universitario A.Gemelli IRCCS, Rome, Italy; d Upper Limb Unit, Shoulder Service, Ospedale Generale Regionale "F.Miulli", Acquaviva delle Fonti, Bari, Italy

**Keywords:** Osteochondroma, Exostosis, Osgood-Schlatter disease, Orthopedic, Pediatric, Athletes

## Abstract

We report a case of a 9-year-old male with anterior knee pain and swelling associated with physical activity. The orthopedic surgeon suspected Osgood-Schlatter’s disease. X-ray and MRI showed an osteochondroma of the tibial tuberosity. The patellar tendon partially inserted on the osteochondroma and the tibial tubercle, causing pain associated with physical activity. We chose a conservative management with temporary rest, local application of ice and NSAIDs. After a few weeks, the boy returned to regular training without pain and our final recommendation was only radiological monitoring of the lesion over the time.

## Introduction

Osteochondromas are common benign bone tumors, usually asymptomatic and detected incidentally, most frequently before the age of 20 years [1]. They often arise from long bones metaphysis. Almost half of them are located around the knee [1]. Distal femur is the preferred site (30% of cases). Tibial osteochondromas account for 15%–20% of cases and most commonly occur in a proximal location [2]. Osteochondromas arising from tibial tuberosity are very rare and there are few cases reported in literature for our knowledge [[3], [4], [5]]. It can manifest clinically with knee pain and swelling mimicking Osgood-Schlatter’s disease (OSD) especially in very young patients. It is necessary to clearly differentiate between the 2 conditions as both are common in adolescents but osteochondromas sometimes require surgery, whereas OSD is generally a self limiting condition.

## Case report

**Fig. 1:**
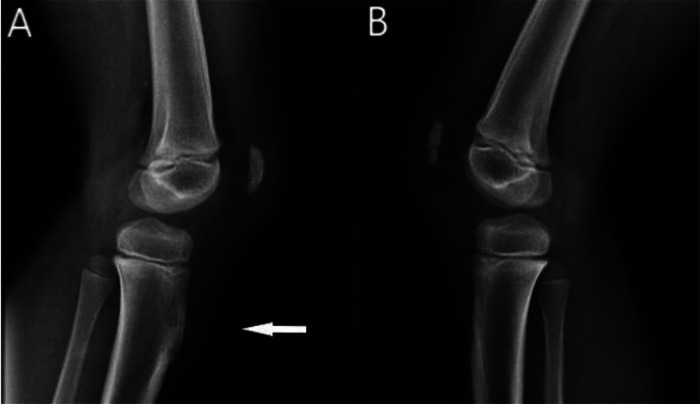
Lateral view knee X-ray. Lef knee (A) showed a bony bump of the tibial tuberosity (arrow). Right knee (B) was completely normal.

**Fig. 2:**
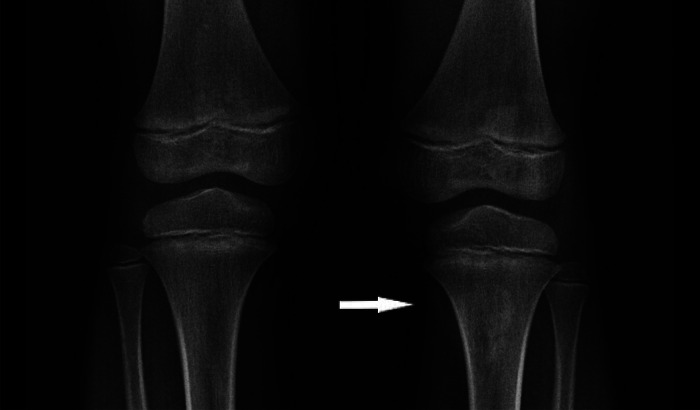
Antero-posterior view knee X-ray. Irregular opacity at the site of the anterior tibial tuberosity of the left knee (arrow) compared to the normal right knee.

**Fig. 3:**
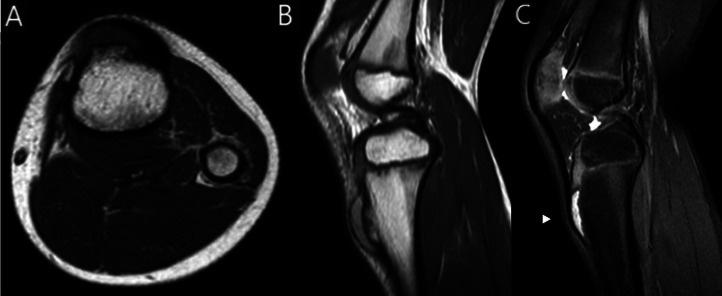
MRI of the left knee. TSE T1w axial (A) and sagittal (B) section showed a protruded bone lesion of the tibial tuberosity with medullary and cortical continuity with the underlying bone. STIR sagittal section (C) better showed the cartilage cup (arrow head) underneath the patellar tendon.

**Fig. 4:**
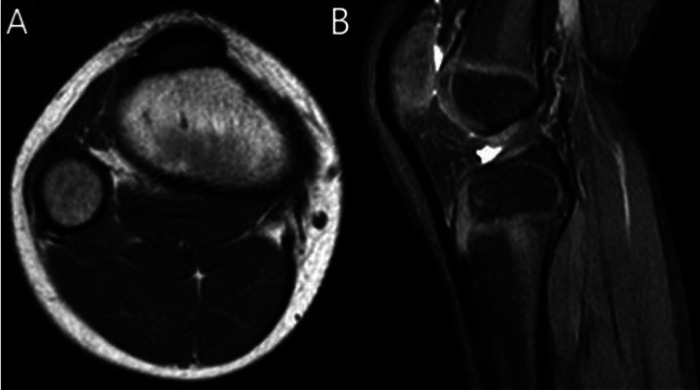
MRI of the right knee. TSE T1w axial (A) and STIR sagittal (B) section of the right side showed normal aspect of the tibial tuberosity.

A 9-year-old male presented with anterior left knee pain. He was a soccer player (training 4 times a week, 3 hours per session) and complained of anterior knee pain associated with physical activity for a year. There was no history of significant previous trauma. On physical examination, he showed swelling on the anterior aspect of his proximal tibia. The swelling was hard in consistency and no painful. Knee range of motion was full but slightly painful on maximum degree of extension and flexion. The orthopedic surgeon suspected an OSD and ordered radiographic imaging. The X-ray showed a bony bump of the tibial tuberosity on the affected knee, however the contralateral was normal (Fig. 1, Fig. 2), in consequence OSD was ruled-out. The radiologist suggested to perform MRI, that showed a protruded bone lesion of the tibial tuberosity with medullary and cortical continuity with the underlying bone. A 4 mm thick cartilage cup, with no calcification was recognized on the bone lesion (Fig. 3, Fig. 4). The diagnosis of osteochondroma was made. The patellar tendon partially inserted on the osteochondroma and the tibial tubercle. Probably his pain was due to patellar tendinopaty. When an osteochondroma is symptomatic, surgical excission should be considered. In literature surgery is recommended ideally after skeletal maturity in order to avoid iatrogenic injury to the growth plate during surgery [4,6], leading to limb length discrepancy. That’s why we chose a conservative management with temporary rest, local application of ice and NSAIDs. After a few weeks, the patient returned to regular training without pain and our final recommendation was only radiological surveillance of the lesion over the time.

## Discussion

Osteochondromas are usually found on long bone metaphysis, near the physis [6,2]. Osteochondromas result from the separation of a fragment of epiphyseal growth plate cartilage. Persistent growth of this cartilaginous fragment and its subsequent enchondral ossification result in a subperiosteal osseous excrescence with a cartilage cap [2]. Their growth is simultaneous with the patient's growth and usually ceases when skeletal maturity is reached [6]. They are seen most often on the distal femur, the proximal tibia and the proximal humerus [2]. These lesions are solitary for about 90% [6]. Osteochondromas may be multiple, when associated with the hereditary multiple exostoses syndrome (HME) [2]. Osteochondromas are most frequently detected before the age of 20 years [1] and are usually asymptomatic. The most common symptom related to osteochondroma is a nontender, painless cosmetic deformity. Additional complications can cause symptoms: osseous deformity, fracture, muscle and neurovascular impingement, overlying bursa formation and malignant transformation (approximately 1% of solitary osteochondromas) [2]. Our patient presented with anterior non tender knee swelling. He had mild pain during maximal flexion and extension, probably as the patellar tendon partially inserted on both the osteochondroma and tibial tubercle.

OSD is one of the most common causes for anterior knee pain in children and adolescents. It is is a traction apophysitis of the tibial tubercle and occurs especially in young athletes. It is caused by repetitive strain of the quadriceps tendon on the secondary ossification center of the tibial tuberosity during the apophyseal maturation stage of the tibial tuberosity, resulting in fragmentation of the tibial tubercle [7]. OSD may present bilaterally in up to 30% of patients [3,5]. It usually presents with anterior knee pain that increases with frequent physical activity. On physical examination, the affected knee may present with tibial tubercle swelling, tender in consistency, and pain, mostly during extension of the knee. Sometimes there is thickening of the patellar tendon [7]. In our patient during the first examination OSD was suspected. This was probably related to the fact that the patient was an athletes and complained of anterior knee pain exacerbated by physical activity for a year. Imaging is crucial to make diagnosis. In our case the standard radiographs was enough to rule out OSD. It showed a well-defined protuberance on the external surface of a bone with cortical and marrow continuity of the lesion and parent bone: this feature is patognomonic of osteocondroma [1]. On the other hand OSD presents with fragmentation of the tibial tubercle. CT and MRI in osteochondroma allow optimal depiction of the cortical and marrow continuity of the lesion and parent bone [2]. MR imaging is the best radiologic modality for evaluating the hyaline cartilage cap, with its low–intermediate signal on T1-weighted images and high signal on T2-weighted images due to its high-water content. A cartilage cap thickness greater than 3 cm in children or 2 cm in adults indicates malignant transformation (secondary chondrosarcoma). Also septal enhancement after gadolinium administration suggests malignant degeneration [1]. MRI imaging also provides information about the lesion effects on surrounding structures (osseous deformity, fracture, muscle impingement, neurovascular impingement, overlying bursa formation and malignant transformation) [2]^.^

What is remarkable in this case report is that the orthopaedic surgeon at the first clinical evaluation did not suspected the possibility of osteochondroma. Other frequent causes of anterior knee pain and swelling associated with physical activity in young patients, expecially athletes [8] are summarized in Table 1.

**Table 1:** OSD main differential diagnosis in young athletes.

Sinding -Larsen-Johansson disease Patellofemoral syndrome Patellar or quadriceps tendinopaty Hoffa fat pad impingement Symptomatic bipartite patella Prepatellar bursitis Stress fracture of the patella

Of course, there are many more possible differential diagnosis of OSD (rheumatologic conditions, malignancy such as osteosarcoma, Ewing sarcoma and synovial tumors, septic arthritis and osteomyelitis): these diseases are rare and usually associated with other symptoms including fever, unintentional weight loss, night pain, pain at rest and erythaema [8].

Benign bone tumors of the tibial tuberosity can accurately emulate OSD. They are very rare. For our knowledge, in literature there is a small number of cases of benign bone tumors mimicking OSD in young patients: 5 cases of solitary osteochondroma [[3], [4], [5]], 5 cases of periosteal chondroma [5,9] and only 1 case of Dysplasia Epiphysealis Hemimelica [5]. The age of these patients (8 males and 3 females) ranged from 7 to thirteen years. Table 2 summarize demographical and clinical characteristics of patients. The limited number of cases don’t allow the statistical analysis of the data.

**Table 2:** Benign bone tumors mimicking OSD with demographical and clinical characteristics of patients.

Souce	Age (years)	Gender	Pain	Final diagnosis
Agaronnik ND^a^	11	M	Positive	Osteochondroma
Balaji G^b^	12	M	Positive	Osteochondroma
Jamshidi K^c^	7	M	Positive	Periosteal chondroma
Jamshidi K^c^	14	M	Positive	Periosteal chondroma
Jamshidi K^c^	12	M	Positive	Periosteal chondroma
Jamshidi K^c^	10	F	Negative	Periosteal chondroma
Jamshidi K^c^	8	M	Negative	Dysplasia Epiphysealis Hemimelica
Jamshidi K^c^	13	F	Negative	Osteochondroma
Jamshidi K^c^	9	M	Negative	Osteochondroma
Jamshidi K^c^	11	M	Negative	Osteochondroma
Vancauwenberghe T^d^	11	F	Negative	Periosteal chondroma

a Agaronnik ND, Landrum M, Wait T, Hogue GD. Osteochondroma of the tibial tubercle masquerading as Osgood-Schlatter disease: a case report. Clin Med Insights Case Rep. DOI: 10.1177/11795476221111771.

b Balaji G, Palaniappan P, Nema S, Menon J. Solitary osteochondroma of the tibial tuberosity mimicking Osgood-Schlatter lesion: a rare cause of anterior knee pain in adolescents: a case report. Malays Orthop J. 2016 Jul;10(2):47-49.

c Jamshidi K, Mirkazemi M, Izanloo A, Mirzaei A. Benign bone tumours of tibial tuberosity clinically mimicking Osgood-Schlatter disease: a case series. Int Orthop. 2019;43:2563-2568.

d Vancauwenberghe T, Vanhoenacker FM, Van Doninck J, Declercq H. Periosteal chondroma of the proximal tibia mimicking Osgood-Schlatter's disease. JBR-BTR. 2013;96(1):30-3.

The aim of this case report is to stress the rule of radiological examinations in young patients with anterior knee pain, helping the orthopedic surgeon achieve diagnosis and choose the proper treatment.

Specifically OSD is generally a self-limiting disease[6]. First-line therapy includes rest and treatment options that alleviate pain and swelling of structures around the tibial tubercle. Local application of ice and NSAIDs can be used for symptom relief. When conservative management fails (about for 10%), surgical intervention (through the excision of the ossicle and/or free cartilaginous material) is considered only after skeletal maturity [7].

Osteochondroma is a benign lesion but can progress in size and lead to complications, such as osseous deformity, fracture, muscle and impingement, overlying bursa formation and malignant transformation. Therefore, it always requires radiological surveillance over the time. Surgery is indicated in case of symptoms from pressure on surrounding structures, cosmetic reasons, uncertain diagnosis and malignant transformation [6,1]. When the tumor is asymptomatic, ideally, surgical excission should be considered only after skeletal maturity in order to avoid iatrogenic injury to the growth plate during surgery [4,6], leading to limb length discrepancy. In our case, symptoms have not been judged sufficiently life-limiting to take this risk. As first line treatment we chose a conservative management with temporary rest, local application of ice and NSAIDs. Symptoms responded well to conservative measures probably because they depended on patellar tendinopaty. Then, we only suggested radiological surveillance of the lesion over the time to monitor its growth and possible occurrence of complications.

## Conclusions

Osteochondroma and OSD typically occur in children and adolescents. OSD usually presents in athletes with anterior knee pain. The pain increases with physical activity. Tibial tuberosity osteochondroma is rare and can clinically mimick OSD in young patients. Whereas OSD is a self-limiting lesion, osteochondroma can grow, lead to complications and require surgery. Although sometimes conservative management can give pain relief, osteochondroma needs always radiological surveillance over the time. For all these reasons in essential to differentiete between these 2 entities. For this purpose, radiological examinations are crucial.

## Ethical approval

Waived.

## Author contributions

All authors participated in conception, analysis, interpretation, drafting, and critical revision of the manuscript.

## Patient consent

Informed written consent was obtained from the patient for publication of the Case Report and all imaging studies. Consent form on record.
